# Comorbidities impact and de-prescribing in elderly with HCV-related liver disease: analysis of a prospective cohort

**DOI:** 10.1007/s11739-021-02741-9

**Published:** 2021-04-28

**Authors:** Anna Licata, Maria Giovanna Minissale, Lydia Giannitrapani, Filippo A. Montalto, Clelia Lombardo, Luigi Mirarchi, Simona Amodeo, Maurizio Soresi, Giuseppe Montalto

**Affiliations:** 1 Internal Medicine and Hepatology, Department of Health Promotion Sciences, Maternal and Infant Care, Internal Medicine and Medical Specialties, PROMISE, University of Palermo Medical School, Piazza delle Cliniche 2, 90127 Palermo, Italy; 2 Instiute for Biomedica Research and Innovation, National Research Council (CNR), 90146 Palermo, Italy

**Keywords:** HCV, Elderly, Co-morbidities, Charlson Index, DDI interactions, De-prescribing

## Abstract

Management for HCV has undergone a notable change using direct-acting antiviral drugs (DAAs), which are safe and effective even in elderly. Here, we define impact of comorbidities, concomitant medication and drug–drug interactions in elder patients with HCV related disease before starting DAAs regimen. We analyzed data of 814 patients prospectively enrolled at our Unit within the web based model HCV Sicily Network. Out of 814, 590 were treated with DAAs and 414 of them were older than 65 years. We divided those 414 in two groups, one including 215 patients, aged between 65 and 74 years, and another with 199 patients, aged of 75 years and over. Charlson Comorbidity Index (CCI) was assessed for each patient; drug–drug interactions (DDI) and de-prescribing process were carried out appropriately. Within 414 patients included, percentage rates of women treated was higher than males, BMI was lower and cirrhosis was frequently reported in patients older than 75 years. Hypertension, diabetes mellitus, dyslipidemia (*p* < 0.0001), prostatic pathologies, kidney disease, gastrointestinal disease (*p* < 0.0001), osteoporosis (*p* < 0.01) and depression were most common co-morbidities. CCI showed lower scores in the first group as compared with the second one (*p* < 0.0001). Among drugs, statins were frequently suspended and anti-hypertensive often replaced. DAAs are useful and effective regardless of disease severity, comorbidities, medications and age. De-prescribing allows a stable reduction of number of medications taken with real improvement of quality of life.

**Supplementary Information:**

The online version contains supplementary material available at 10.1007/s11739-021-02741-9.

## Introduction

Hepatitis C virus infection affects many individuals worldwide and its prevalence increases with aging [1–3] . Accordingly, most of the actually infected subjects are old, being born between 1945 and 1965 [4]. However, HCV morbidity and mortality rates could be underestimated because of concomitant disease. Clinical heterogeneity and genotypic mutation capacity are probably involved in the high rate of chronicity of the infection [5], and due to the high prevalence of the disease in elderly, older patients constitute the main “reservoir” of the virus. Marcellin et al., investigating risk factors responsible of fibrosis progression in chronic hepatitis C, showed that age, alcohol consumption and male sex were associated with a faster course of disease [6]. Thus, age in elderly represents an important factor predicting disease progression [2–8].

For many years, interferon (IFN) and ribavirin were the only antiviral therapies available for treatment of HCV infection. However, due to wide range of side effects, these drugs were often contraindicated, especially in elderly with comorbidities [9, 10]. Direct acting antiviral agents (DAAs) have radically changed the clinical scenario of HCV management, even in the elder patients, regardless of stage of liver disease and IFN ineligibility [11, 12]. Safe treatment of patients aged of 65 years and over suggests that sustained viral response (SVR) rates are independent of age, and the contraindication eventually comes from drug interactions with medications taken for comorbidities [13, 14].

Aging, is usually accompanied by increasing presence of co-morbidities, such as hypertension, diabetes, cardiovascular and kidney diseases, which are causes of death [15]. Accordingly, the wide number of medications taken and, their interactions with DAAs, are crucial in the therapeutic choice of DAAs regimen. Load of comorbidities of elder HCV patients could be assessed by different scoring system, as Charlson Comorbidity Index (CCI) [16]. Searching DAAs–drug interactions could be achieved by Liverpool Interaction Checker at: www.hep-druginteractions.org [17]. However, eliminating or reducing certain medication, not only in the elderly patient, represents an optimal strategy to decrease inappropriate use of drugs. “De-prescribing” process is the most effective way to reduce the risk of adverse events from drug–drug interactions [18, 19].

Therefore, aiming to evaluate the impact of co-morbidities in patients of 65 years and over, affected by HCV-related liver disease, we analyzed our prospective cohort including 590 patients who already achieved viral response and are still in follow-up. Furthermore, to eliminate potential drug interactions, we checked all medications taken and to maximize the efficacy of antiviral therapy, avoiding any treatment interruption or onset of adverse events, we properly start de-prescribing process.

## Methods

### Study cohort

In this prospective cohort study, we included patients treated for HCV-related liver disease from April 2015 to December 2019. Data were collected within the web-based model HCV Sicily Network [20]. For all, we collected clinical, laboratory and anthropometric parameters. Data regarding HCV genotype and viral load, HBV and HIV co-infections were assessed, as well.

Laboratory tests and visits were performed at the time of inclusion and every 4 weeks after starting antiviral therapy and finally 12 weeks after the end of the treatment, monitoring hemoglobin, platelets, ALT, albumin, serum glucose, serum creatinine, blood urea nitrogen and INR. Within 6 months preceding antiviral therapy prescription, patients enrolled were assessed by liver ultrasound. In those with cirrhosis, an endoscopy to search for esophageal varices was performed. Liver stiffness of all included patients was measured at inclusion; SVR (HCV-RNA < 15 IU/ml) was assessed at the end of therapy (8 vs. 12 weeks). All patients, after clinical assessment were candidates to antiviral regimen according to AIFA criteria for DAAs prescription (www.aifa.gov.it/web/guest/-/registri-aifa). Treatment schedule, was chosen based on the viral HCV genotype, staging of liver disease and previous antiviral treatment [1].

### Assessment of comorbidity, drug–drug interaction and de-prescribing

At inclusion and before DAA prescription, all patients were searched for the associated co-morbidities and medications taken. Comorbidity evaluation was performed through the Charlson Comorbidity Index (CCI), a score system useful for the clinical decision-making process, which allows to evaluate the total comorbidity load present in each patient. For each comorbidity present, a numeric score ranging from 1 to 6 was assigned according to their effect on mortality and the total score estimates survival at 10 years [16].

After recording the therapies taken for comorbidities, the presence of potential drug–drug interactions, which could interfere with antiviral regimen was assessed by Liverpool Interaction Checker at website: https://www.hep-druginteractions.org. Potential drug–drug interactions were collected and coded with different risk ranging from 0 (interaction has not been assessed), 1 (no clinically significant interaction expected), 2 (potential interaction that may require monitoring) up to 3 (co-administration is not recommended or contraindicated). Replacement, closely monitoring and "de-prescribing" of the drug, were evaluated in patients belonging to category 2 and 3 [21–23].

De-prescribing is a systematic identification process and “discontinuation” of drugs or drug regimens in circumstances in which obvious or potential negative effects exceed current and/or potential benefits, taking into account the goals of care, the life expectancy, values and preferences of the individual patient [23]. This process has been carried-out through the revision of all medications taken from the patients, assessment of risk/benefit of each drug and suspension/replacement of that medication which potentially could interact with antiviral therapy. Suspension and/or replacement of drugs have been closely carried out, through the use of diaries compiled by the patient (e.g., reporting blood pressure values every day if the suspended drug was an anti-hypertensive).

The study was conducted according to the Declaration of Helsinki. The ethics committee of the University Hospital AOUP Policlinico “P.Giaccone” approved the analysis of anonymous patient data; each included patient signed written informed consent.

### Statistical analysis

Data with normal distribution were expressed as mean and standard deviation (SD), otherwise as median and range and analyzed, respectively, with Student’s *t* or Mann Whitney *U* Test. The difference between the frequencies was assessed with the Fisher exact test or with the chi square test. The results were considered significant if *p* < 0.05.

## Results

### Study population and baseline characteristics

Given a total of 814 patients followed at our Internal Medicine and Hepatology Unit and included in the HCV Sicilian Network, those already treated with antiviral therapy were 590. Of these 414 (70%), who were over 65 years of age, represented our study population. Patients included were divided in two groups: a group with age between 65 and 74 years and another with an age over than 75 (≥ 75). Demographic, clinical and laboratory features are shown in Table 1. Percentage rate of woman treated was higher in both groups. BMI was significantly lower in older patients (*p* < 0.00001). Platelets count was lower in the group of older patients (*p* < 0.05) and accordingly cirrhosis, whereas chronic hepatitis was significantly more frequent in the first group (Table 1).

**Table 1:** Baseline features of patients with HCV-related liver disease treated with DAA, stratified by age

	65–74 years *n* = 215 (52)	≥ 75 years *n* = 199 (48)	*p* <
Male gender	84 (39)	93 (46.7)	ns
BMI (Kg/m^2^)	26.8 ± 3.8	25.4 ± 3.6	0.0001
HCVRNA > 800.000 U/ml	127 (59.1%)	121 (60.8%)	ns
Genotype			
1b	169 (78.6)	167 (84)	ns
1a	7 (3.2)	2 (1)	ns
2	27 (12.6)	27 (13.6)	ns
3	3 (1.4)	0	ns
4	9 (4.2)	3 (1.4)	ns
ALT (IU/L)	68.4 (9–331)	59.5 (11–205)	ns
PTL/mmc	169.705 (320–414000)	158.525 (370–880000)	0.05
Bilirubin (mg/dl)	0.9 ± 0.6	0.8 ± 0.5	ns
Albumin (gr/dl)	3.9 ± 0.4	3.8 ± 0.4	ns
INR	1.07 ± 0.24	1.06 ± 0.21	ns
Fibroscan (KPa)	11 (2.9–70.6)	11.9 (3.2–74.6)	ns
Stage of disease			
Chronic hepatitis	141 (65.6)	100 (50.3)	0.002
Cirrhosis	74 (34.4)	89 (49.7)	0.05
HCC	3 (1.9)	10 (5)	ns
Presence of varices	34 (45.9)	49 (55)	ns
Child–pugh class			
A	70 (94.6)	86 (96.6)	ns
B	4 (5.4)	3 (3.4)	ns
Previous treatment:			
Naive	106 (49.3)	120 (60.3)	ns
Experienced	109 (51.7)	79 (39.7)	0.03
Co-morbidities			
Arterial hypertension	147 (68.4)	152 (76.4)	ns
Cardiomyopathy	8 (3.7)	11 (5.5)	ns
Diabetes mellitus	42 (19.5)	45 (22.6)	ns
Dyslipidemia	14 (6.5)	22 (11)	0.0001
Kidney disease	7 (3.3)	12 (5)	ns
Prostatic hypertrophy	18 (8.4)	27 (13.6)	ns
Gastro-intestinal disease	80 (37.2)	121 (60.8)	0.0001
Thyroid disease	33 (15.3)	20 (10)	ns
Respiratory disease	12 (5.6)	9 (4.5)	ns
Osteoporosis	13 (6)	29 (14.6)	0.01
Depression	22 (10.2)	8 (4)	
Charlson Comorbidities Index (score)			
3–5	131 (61)	74 (37)	0.0001
6–8	82 (38)	105 (53)	0.005
9–11	2 (1)	20 (10)	0.0001

### Efficacy and safety profile of DAAs in elderly

Based on the obtained results from clinical and laboratory tests, the choice of the most suitable DAAs treatment schedule was made for each patient. In both groups, the most prescribed regimen was the association of sofosbuvir/velpatasvir, followed by glecaprevir/pibrentasvir. Prescription of elbasvir/grazoprevir was more common in the group of patients over 75, as compared with those 65–74 (*p* < 0.004). At the end of therapy, HCV-RNA viral load was performed to verify SVR, which was 96.3% in the first group and 97.5% in the second.

Eight out of 215 (3.7%) did not reach SVR: four patients did not finish treatment, two drop-out and two due to side adverse effects (SAEs). Regard patients with SAEs they were both women: one showed severe thrombocytopenia at 4 weeks from starting therapy with PLTs less than 3000/mmc and the other showed edema and redness of the left upper arm after 7 weeks; one patient did not respond to therapy and three relapsed. Within the second group, 5 out of 199 (2.5%) did not reach SVR: three did not finish therapy due to side effects (two patients for progression of renal damage with increase of serum creatinine, one due to increase of alanine-aminotrasferases more than 7 times ULN); one patient did not respond to therapy and another patient relapsed (Supplementary Table).

### Assessment of Charlson Comorbidities Index, comparison by age groups

For all patients, comorbidities and concomitant medication were analyzed. Given the two groups, patients were divided according to the number of concomitant disease (Table 2).

**Table 2:** Comparison of Charlson Index in patients with HCV-related liver disease according to age groups

Charlson Comorbidity Index	65–74 years *n* = 215 (52%)	≥ 75 years *n* = 199 (48%)	*p* <
Score			
3	29 (13.5%)	0	0.0001
4	72 (33.5%)	40 (20.1%)	0.003
5	30 (13.9%)	34 (17.1%)	ns
6	46 (21.4%)	51 (25.6%)	ns
7	28 (13.1%)	38 (19.2%)	ns
8	8 (3.7%)	16 (8%)	ns
9	0	10 (5%)	0.001
10	2 (0.9%)	8 (4%)	0.001
11	0	2 (1%)	ns

**Fig. 1:**
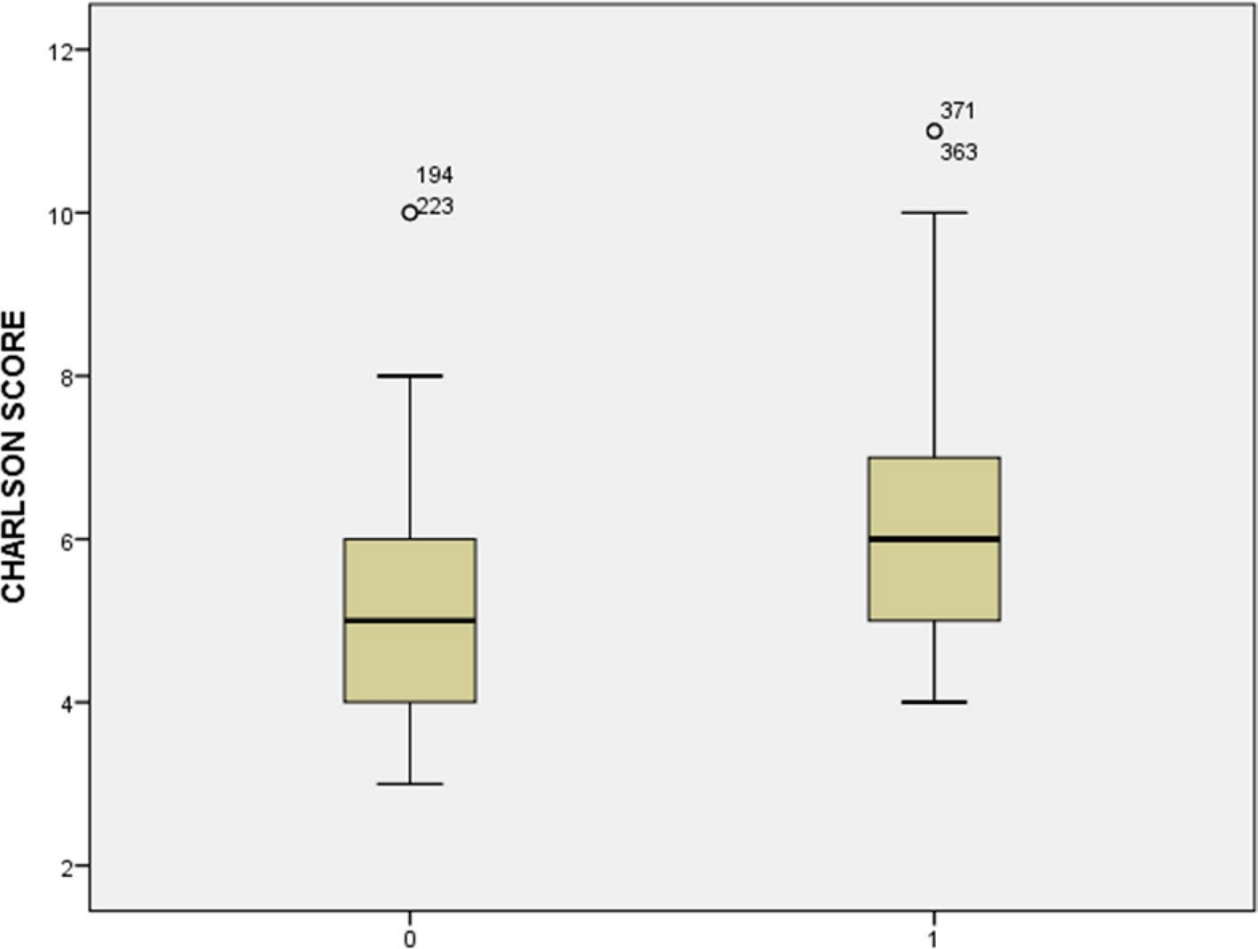
Analysis of differences within CCI in patients younger and older than 75 years: mean number of comorbidities was 5 (3–10) in younger and 6 (5–11) in older than 75

Assessment of Charlson Comorbidity Index (CCI) in the study population showed lower scores in the first group of patients aged between 65 and 74, whereas higher scores prevail in the second. Sixty-one percent of the patients in the first group presented an index score less than 5, whereas in the second group, more than 50% showed a score of 6–8. A comparison between groups showed difference statistically significant between scores (3–5) *p* > 0.0001, 6–8 (*p* < 0.005), 9–11 (*p* < 0.0001). Differences within CCI in patients lower than 75 years showed that the mean number of comorbidities was 5 (3–10), whereas in patients with age over 75 was 6 (4–11) (Fig. 1).

Arterial hypertension was frequently present and the majority of patients undergo combined therapies. Patients with dyslipidaemia receiving lipid lowering drugs were 6.5% in the first group and 11% in the second one, being statins and ezetimibe/omega-3 the most administered drugs (*p* < 0.0001). Type 2 diabetes mellitus was treated in 19.5% in the first group and 22.6% in the second; in both groups, more than 50% of patients were treated with insulin, whereas metformin was the most commonly prescribed anti-diabetic drug. Osteoporosis therapy was administered in 6% within the first group and 14.6% within the second (*p* < 0.01). Therapies for gastrointestinal pathologies showed a high frequency in patients over 75 years (*p* < 0.0001), being proton-pump inhibitors the mostly taken medications.

### Drug–drug interactions and de-prescribing

As shown in Table 3, the number of medications taken by each patients before starting antiviral therapy was higher for those of age older than 75 years, and this difference was statistically significant (*p* < 0.0001).

**Table 3:** Number of medications taken by each patients with chronic HCV-related liver disease stratified by age and de-prescribing after checking DDI interaction

Medications	65–74 years *n* = 215 (52%)	≥ 75 years *n* = 199 (48%)	*p* <
0–3	137 (63.7%)	85 (42.7%)	0.0001*
4–7	68 (31.6%)	101 (50.8%)	
8–11	10 (4.7%)	13 (6.5%)	

De-prescribing	65–74 *n* = 215 (52%)	≥ 75 *n* = 199 (48%)	*p*
0–3	141 (65.6)	91 (42.7%)	0.0001
4–7	66 (30.7%)	99 (50.8%)	
8–11	8 (3.7%)	9 (6.5%)	

^*^ρ = 0.2

After checking drug–drug interaction at Liverpool website for each patient, we start de-prescribing process. We found that drugs frequently suspended due to interaction with antivirals were within the second group (> 75 years) as compared with the first (*p* < 0.0001). One drug was suspended or replaced in 20.7% of patients (19.1% in the first group and 22.6% in the second group). A drug was suspended in 13% of patients in the first group and 15.6% in the second group. The most frequently suspended drugs due to interactions with antivirals were statins (21.4% vs. 35.5%), proton pump inhibitors (21.4% vs. 12.9%), bisphosphonates/vitamin D (13% vs. 15.6%), and anti-hypertensive drugs (0 vs. 12.9%). Anti-hypertensive drugs (57.2% vs. 44.4%) followed by statins (21.4% vs. 22.2%) in both groups were the most replaced with another medication of the same class (6,5% in the first group and 9% in the second) due to moderate or severe interaction with antiviral therapy, and thus, preventing the reliable occurrence of any adverse event leading to stopping antivirals (Table 4). Suspension and/or replacement of drugs have been closely carried-out through a close monitoring of the patients during the monthly visits. None of the suspended or replaced drug have been re-entered within the medications taken at the end of antiviral therapy. Sometimes only dosage of anti-hypertensive drugs have been increased with the purpose of the reaching therapeutic range, when antiviral therapy have been completed.

**Table 4:** Analysis of drugs suspended or replaced for patients with HCV-related liver disease before starting DAAs, stratified by age

De-prescribing	65–74 years *n* = 215 (52%)	≥ 75 years *n* = 199 (48%)	*p* <
Total: 86 (20.7%)	41 (19.1%)	45 (22.6%)	ns
Suspension 1 drug	28 (13%)	31 (15.6%)	ns
Bisphosphonates/vitamin D	11 (39.3%)	9 (29%)	ns
Statins	6 (21.4%)	11 (35.5%)	ns
Anti-hypertensive	0	4 (12.9%)	0.05
PPI	6 (21.4%)	4 (12.9%)	ns
Other	5 (17.9%)	3 (9.7%)	ns
Substitution 1 drug	14 (6.5%)	18 (9%)	ns
Statins	3 (21.4%)	6 (33.3%)	ns
Anti-hypertensive	8 (57.2%)	8 (44.4%)	ns
Other	3 (21.4%)	4 (22.2%)	ns

## Discussion

Availability of DAAs allowed elder people to access antiviral treatment safely, with response rates of more than 95%, regardless of comorbidities. Thus, a careful assessment of the patient’s geriatric status is mandatory before starting DAAs regimen.

In our study, we found that among 414 elder patients studied, percentage rates of women treated was higher than males, BMI was lower and cirrhosis was frequently reported in patients older than 75 years. CCI showed lower scores in the first group as compared with the second, in which scores were significantly higher (*p* < 0.0001). Arterial hypertension was the comorbidity most frequent, followed by type 2 diabetes, dyslipidemia and osteoporosis; classes of drugs most administered were statins, which were also the most frequently suspended, following by proton pump inhibitors, bisphosphonates/vitamin D; anti-hypertensive drugs were the most replaced medications.

Percentage of elderly patients with HCV infection treated was very low before the introduction of the DAAs [13] and this was mainly due to the high number of comorbidities, which constituted an absolute contraindication in the era of IFN/RBV treatment [10]. However, with the introduction of DAAs regimen, it was possible to increase the number of elder patients treated [24], as was shown in our cohort, as well.

As already noted, clinical features of our cohort regarding genotype and stage of liver disease confirm the epidemiological data of HCV infection in Italy [25, 26], thus patients started DAAs regimens according to AIFA criteria, in force at the time of prescription. In general, all treatment schedules were well tolerated; no patient died during antiviral therapy, nor in the following 6 months. Adverse events within the entire cohort were reported only in five cases, two in the first group and three in the second as described above; none of them was correlated to aging. There were no substantial differences in terms of SVR between the two groups, confirming results obtained from other cohorts [13, 14, 24]. In patients over 75 years, an increased number of medication intake and reduced glomerular filtrate (GFR) prevailed. In this population, elbasvir/grazoprevir, which is indicated for patients with reduced GFR, was the most prescribed DAAs as compared with patients younger (65–74 years). Moreover, among DAAs, it represents the medication with fewer drug interactions on Liverpool website and thus useful for older patients on poly-therapy.

Low BMI in these patients denotes that a mild sarcopenia, typical of the elderly cirrhotic patient, was frequent [27]. Mild sarcopenia is consistent with advanced age, cirrhosis and osteoporosis and accordingly these comorbidities were features typically of our cohort, in which patients older than 75 years had a BMI lower than those aged 64–75 years.

**Fig. 2:**
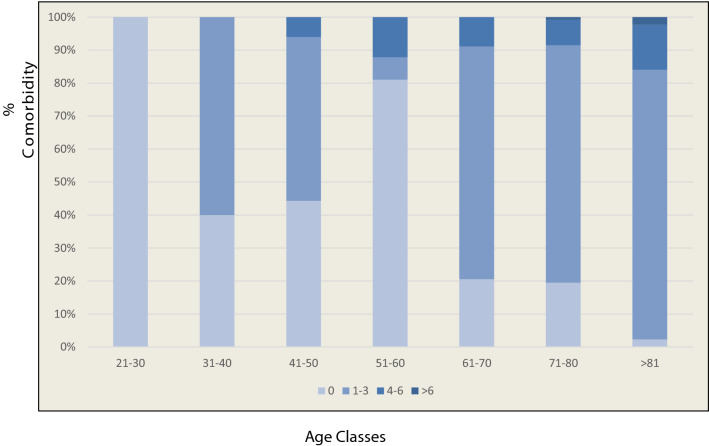
Analysis of percentages of comorbidities 1–3, 4–6, > 6, according different age classes (years)

In elderly patients, comorbidities are numerous, actively contribute to the progression of HCV-related disease and further decreases quality of life [2]. We calculated the Charlson Comorbidity Index [16], which predicts the 1-year mortality for a comorbid condition and how aggressively to treat it. More than half (61%) of our patients in the group of 65–74 years had a CCI less than 5, whereas more than 50% of the patients aged more than 75 years had a score between 6 and 8 (Fig. 2). Thus, as it has showed by Ruzicka et al. [28], increasing age is associate with a major number of diseases.

The concept of comorbidity is usually associated with the poly-therapy, and elderly patients are currently being prescribed with a median of seven medications, which often causes more harm than benefit, and emphasizes the importance of “de-prescribing” [29]. This process consistent with tapering, stopping, discontinuing, or withdrawing drugs, with the goal of managing polypharmacy, improve the appropriateness of prescriptions and clinical outcomes. In our study, one drug was suspended or replaced in 20.7% of patients, stopped in about 15%. The most frequently suspended and/or replaced drugs were: bisphosphonates/vitamin D, proton-pump inhibitors, statins and anti-hypertensive drugs. In particular, arterial hypertension was the most frequent comorbid condition in both groups, since it perfectly correlates with other published studies [30, 31]. The need of anti-hypertensive therapy, with one or more medication, in the elderly is rigorously associated to the risk of complications due to atherosclerosis and cardiac abnormalities, as increasing of left ventricular mass, which represent the major risk factor for transient ischemic attack and stroke [32, 33]. Given such potential drug interactions with DAAs, needed medications to avoid above complications should be adequately replaced. Accordingly, the highest number of substituted drugs to avoid harmful events leading to stopping antivirals have been found in hypertensive patients, within the following category of drugs: calcium-channels blockers, heart failure agents and statins. As regards to osteoporosis, de-prescribing of vitamin D/bisphosphonate was frequently performed not because of drug interactions, but aiming to improve compliance to antiviral therapy in patients on polytherapy, sometimes following the Beers criteria, as well [34].

The new DAAs, although safe and effective, present an important number of drug interactions, which if not properly evaluated could compromise the outcome of the treatment and, therefore, the patient's health, as also underlined in the observational cohort of PITER study [35, 36]. In our study, no significant adverse events caused by drug interactions have been detected. This was probably due to the careful assessment of drug–drug interactions before starting antiviral therapy, followed by the close clinical monitoring. Furthermore, following the end of antiviral treatment, patients usually continued to practice therapy as it has been prescribed, before starting DAAs, with an overall reduction in the number of drugs taken, in accordance with pharmaco-economics principles and decreasing health costs.

In conclusion, advanced age should not be considered a barrier to antiviral treatment, as the safety profile found was optimal as compared with the high number of comorbidities and concomitant medications. In our study, we have shown that DAAs determines a high rate of response in elderly population, regardless of disease severity and comorbidity. Overall, de-prescribing in these complex patients constitutes a step forward in the prevention of adverse events, representing a real improvement for the quality of life, as well.

## Supplementary Information

Below is the link to the electronic supplementary material.

Supplementary file1 (DOCX 15 KB)
