# Supplementary material for: Comorbidities impact and de-prescribing in elderly with HCV-related liver disease: analysis of a prospective cohort
Source: Intern Emerg Med. 2021 Apr 28;17(1):43–51. doi: 10.1007/s11739-021-02741-9 (PMC8841322; doi:10.1007/s11739-021-02741-9)
Supplement: Supplementary file 1 — Supplementary file1 (DOCX 15 KB) [file 11739_2021_2741_MOESM1_ESM.docx]

**Supplementary Table** — DAAs regimen, efficacy and adverse events of treated patients, stratified by age.

|  | **65 – 74 years old**  **n = 215 (52 %)** | **≥75 years old**  **n = 199 (48 %)** | ***P<*** |
| --- | --- | --- | --- |
| **Sofosbuvir/Velpatasvir**  **Sofosbuvir/Ledipasvir**  **Sofosbuvir/Ledipasvir + RBV**  **Glecaprevir/Pibrentasvir**  **Elbasvir/Grazoprevir**  **Ombi/Parit/Rit /Dasabuvir**  **Ombi/Parit/Riton/Das+ RBV**  **Sofosbuvir Daclatasvir + RBV** | 63 (29,3 %)  23 (10,7 %)  25 (11,6 %)  45 (20,9 %)  18 (8,4 %)  14 (6,5 %)  12 (5,6 %)  15 (7 %) | 61 (30,6 %)  20 (10,1 %)  21 (10,6 %)  32 (16,1 %)  30 (15,1 %)  11 (5,5 %)  10 (5 %)  14 (7 %) | ns  ns  ns  ns  **0.004**  ns  ns  ns |
| **Use of RBV** | 48 (22.3%) | 41 (20.6) | ns |
| **Treatment duration**  **- 8 Weeks**  **- 12 Weeks**  **- 24 Weeks** | 44 (20.4)  149 (69.3)  21 (9,8) | 29 (14.6)  142 (71.3)  21 (10.6) | ns  ns  ns |
| **No SVR** | 8 (3,7 %) | 5 (2,5 %) | ns |
| **Drop-out/AEs** | 4 (1,8 %) | 3 (1,5 %) | ns |
